# Utility of unidimensional and functional pain assessment tools in adult postoperative patients: a systematic review^☆^

**DOI:** 10.1016/j.bja.2021.11.032

**Published:** 2022-01-05

**Authors:** Reham M. Baamer, Ayesha Iqbal, Dileep N. Lobo, Roger D. Knaggs, Nicholas A. Levy, Li S. Toh

**Affiliations:** 1 Division of Pharmacy Practice and Policy, School of Pharmacy, University of Nottingham, Nottingham, UK; 2 Pharmacy Practice Department, Faculty of Pharmacy, King Abdul-Aziz University, Jeddah, Saudi Arabia; 3 Nottingham Digestive Diseases Centre, National Institute for Health Research, Nottingham Biomedical Research Centre, Nottingham University Hospitals NHS Trust and University of Nottingham, Queen's Medical Centre, Nottingham, UK; 4 MRC Versus Arthritis Centre for Musculoskeletal Ageing Research, School of Life Sciences, University of Nottingham, Queen's Medical Centre, Nottingham, UK; 5 Pain Centre Versus Arthritis, University of Nottingham, Nottingham, UK; 6 Department of Anaesthesia and Perioperative Medicine, West Suffolk Hospital NHS Foundation Trust, Bury St. Edmunds, UK

**Keywords:** COnsensus-based Standards for the selection of health Measurement INstruments (COSMIN), functional pain assessment tool, pain scores, postoperative pain, tool utility, unidimensional pain assessment

## Abstract

**Background:**

We aimed to appraise the evidence relating to the measurement properties of unidimensional tools to quantify pain after surgery. Furthermore, we wished to identify the tools used to assess interference of pain with functional recovery.

**Methods:**

Four electronic sources (MEDLINE, Embase, CINAHL, PsycINFO) were searched in August 2020. Two reviewers independently screened articles and assessed risk of bias using the COnsensus-based Standards for the selection of health Measurement INstruments (COSMIN) checklist.

**Results:**

Thirty-one studies with a total of 12 498 participants were included. Most of the studies failed to meet the methodological quality standards required by COSMIN. Studies of unidimensional assessment tools were underpinned by low-quality evidence for reliability (five studies), and responsiveness (seven studies). Convergent validity was the most studied property (13 studies) with moderate to high correlation ranging from 0.5 to 0.9 between unidimensional tools. Interpretability results were available only for the visual analogue scale (seven studies) and numerical rating scale (four studies). Studies on functional assessment tools were scarce; only one study included an ‘Objective Pain Score,’ a tool assessing pain interference with respiratory function, and it had low-quality for convergent validity.

**Conclusions:**

This systematic review challenges the validity and reliability of unidimensional tools in adult patients after surgery. We found no evidence that any one unidimensional tool has superior measurement properties in assessing postoperative pain. In addition, because promoting function is a crucial perioperative goal, psychometric validation studies of functional pain assessment tools are needed to improve pain assessment and management.

**Clinical trial registration:**

PROSPERO CRD42020213495.

**Editor's key points**

• Well validated assessment tools are essential for measuring postoperative pain intensity and impact
• This systematic review shows that despite many tools available, evidence regarding their validity or reliability is scarce.
• After surgery, the Visual Analogue Scale (VAS) showed the highest error rate in general and was the least preferred compared to the 0-10 Numerical Rating Scale (NRS).
• Importantly statistically significant changes in VAS or NRS do not necessarily indicate clinically important changes, and NRS cut-off points used by healthcare professionals to determine acute pain severity do not always reflect patients' desire for analgesics.

Patients experience acute pain after surgery as a result of tissue damage and inflammation at the operation site.^1^, ^2^, ^3^ Careful assessment of pain using a valid and reliable tool^4^ is the first step towards a rational choice of analgesic therapy,^5^ which is essential for ensuring patient comfort, mobility, and satisfaction and reducing healthcare costs.^6^ The most commonly used tools for the assessment of postoperative pain are unidimensional and assess only pain intensity.^4^ These include the visual analogue scale (VAS),^7^ numerical rating scale (NRS),^8^ verbal rating scale (VRS),^9^ sometimes referred to as the verbal descriptor scale (VDS),^10^ and faces pain scales (FPS).^11^ They are quick to administer and do not encroach on the time required for usual care.^12^

Despite their extensive use, the reliance on these unidimensional tools as the sole approach to measuring pain is currently insufficient as the cut-off points commonly used by healthcare providers do not reflect the patient's desire for additional analgesics.^13^^,^^14^ Furthermore, patients have reported difficulties in describing the complexity of their pain experience by a single numerical value, descriptive words, or as a mark on a line.^12^ Striving to lower pain intensity scores to zero as suggested by the ‘Pain as the 5th Vital Sign’ campaign has not improved pain outcomes,^15^, ^16^, ^17^ and resulted in increased opioid analgesic use in the post-anaesthesia care unit (PACU).^17^ Furthermore, Vila and colleagues^18^ highlighted the potential hazard associated with a pain score-based treatment algorithm in increasing the prevalence of sedation-related side-effects by more than twofold. Treating pain as the fifth vital sign has been abandoned now as it may have contributed to the current US opioid epidemic.^19^^,^^20^

Restoration of function by allowing the patient to breathe, cough, ambulate, and turn in bed is important for postoperative pain relief.^21^^,^^22^ Therefore, assessing the functional impact of pain, which includes patient-centred objective assessment by a healthcare provider who judges if the pain prevents the patient from performing activities that help accelerate recovery, could be an appropriate alternative to achieve better pain assessment.^23^ Hence, options to treat pain will be used to maximise functional capacity, rather than striving to reduce the patient's postoperative pain score to below a specified numerical value.^4^^,^^20^

Despite being used widely, the validity, reliability, and utility of unidimensional pain assessment tools for postoperative patients have not been reviewed systematically. The aim of this systematic review was to appraise the available evidence concerning the measurement properties of different unidimensional and functional pain assessment tools when used to assess postoperative pain in hospitalised adults.

## Methods

We performed this systematic review according to COnsensus-based Standards for the selection of health Measurement INstruments (COSMIN) (http://www.cosmin.nl/) guidelines, and reported it according to the Preferred Reporting Items for Systematic Reviews and Meta-Analysis (PRISMA) statement guidelines.^24^

### Search strategy

We performed a systematic search of the MEDLINE, Embase, PsycINFO (all via OVID) and CINAHL (via EBSCOhost) databases from their inception to August 2020. Our search strategy consisted of four search concepts: (1) measurement properties or outcome terms, (2) pain assessment tool terms, (3) acute postoperative pain, and (4) limits (English language or English translation, human adults ≥18 yr old). We combined the first three using the Boolean operator AND, which works as a conjunction to narrow the search to include our specific three search concepts resulting in more focused results. This was then combined with the result string of the fourth concept to limit the results. We performed these steps separately for each pain assessment tool. We carried out backward citation tracking as well by checking the reference lists from eligible studies. The comprehensive search strategy used is provided in Supplementary material, Appendix S1.

### Inclusion criteria

We included any of the following pain measurement tools to assess acute pain in hospitalised adult patients from all surgical specialties: unidimensional pain assessment tools (including the numerical pain rating scale, VRS, VAS, faces scales [Wong-Baker FACES, Faces Pain Scale – Revised]), and functional pain assessment tools included any tool that helps assess acute pain based on its interference with functional activity, including walking, breathing, turning in bed, and coughing. Included functional pain assessment tools could be used objectively by the clinician or when self-reported by patients.

We included instrument validation or instrument evaluation types of studies. Any studies that included at least one or more of the instruments to evaluate postoperative pain and assessed at least one of the nine measurement properties identified by COSMIN taxonomy: internal consistency, test–retest reliability, measurement error, content validity, structural validity, construct validity, hypothesis testing, cross-cultural validity, criterion validity, and responsiveness were considered (Appendix S2). In addition, we included any study that evaluated any of the specified additional outcomes of the tools, including feasibility, interpretability, and desire for analgesia.

### Exclusion criteria

We excluded abstracts, editorials, reviews, and studies that included paediatric or adolescent populations, or sedated, mechanically ventilated and critically ill patients.

### Selection of articles

**Fig 1:**
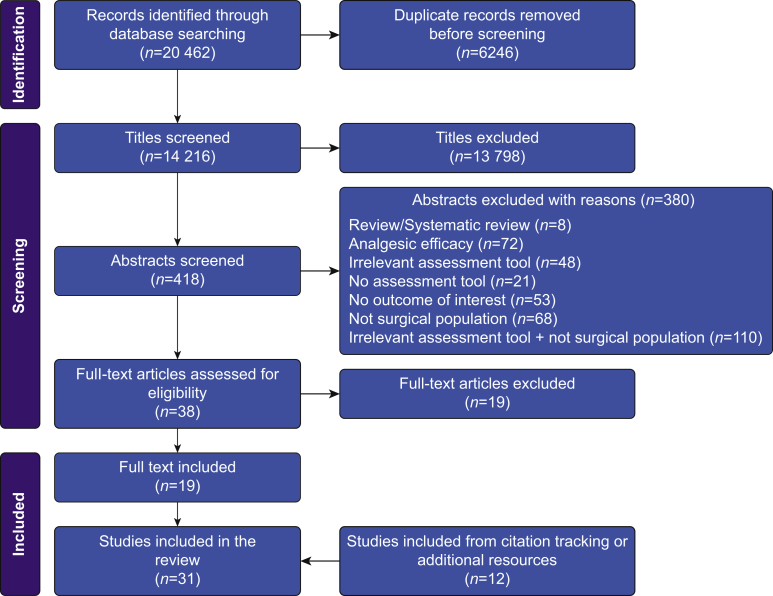
PRISMA diagram. PRISMA, Preferred Reporting Items for Systematic Reviews and Meta-Analyses.

After our database search, we collated and uploaded all identified citations to EndNote X9 (Clarivate Analytics, Philadelphia, PA, USA) and removed duplicates. The identified studies were uploaded to Rayyan QCRI online software.^25^ Two reviewers (RMB and AI) independently applied the inclusion criteria to the titles, then to relevant abstracts. Afterwards, we thoroughly examined potentially eligible full texts for inclusion. We documented the full search results in the PRISMA flow diagram (Fig. 1). Excluded studies and the reasons for their exclusion are provided in Appendix S3.

### Data extraction

One reviewer (RMB) extracted data from the included full-text articles, with the extraction verified by a second reviewer (AI). The two reviewers resolved any disagreements through discussion, or consultation with other reviewers (RDK, LST, or DNL) when necessary. The extracted data included specific details about the assessment tool used, country, language of scale administration, study design, patient characteristics, surgical procedure, the specific measurement properties assessed, outcomes related to the review question and objectives, and the main statistical analysis.

### Assessment of methodology

Two independent reviewers (RMB and AI) critically appraised the methodological quality of studies looking at feasibility and interpretability using a modified version of the Newcastle–Ottawa Scale^26^ (Appendix S4). For validation studies, we assessed the quality using the COSMIN criteria for methodological quality.^27^, ^28^, ^29^ We included three phases in the assessment of each measurement property. First, we assessed the risk of bias, which pertains to methodological quality in each study: very good, adequate, doubtful, or inadequate quality was assigned to each study. Second, we related the results to a measurement property rated against criteria for ‘sufficient measurement properties’, and the results were classified as sufficient, insufficient, or indeterminate (Appendix S5). Third, we combined the results from each study and graded the quality of evidence for each pain assessment tool. A summary of the scoring criteria and appraisals is provided in (Appendices S6 and S7).

### Protocol registration

The protocol was registered (No. CRD42020213495) with the PROSPERO database and can be accessed at https://www.crd.york.ac.uk/prospero/display_record.php?RecordID=213495.

## Results

The search identified 14 216 potential studies after removal of duplicates. After reviewing the titles, we excluded 13 798 for irrelevance and another 380 after abstract screening. Of the 38 remaining studies, we excluded 19 after examination of the full texts against the inclusion criteria (Appendix S2). An additional 12 studies were identified through searching the bibliography of eligible studies, so a total of 31 studies^2^^,^^3^^,^^6^^,^^13^^,^^30^, ^31^, ^32^, ^33^, ^34^, ^35^, ^36^, ^37^, ^38^, ^39^, ^40^, ^41^, ^42^, ^43^, ^44^, ^45^, ^46^, ^47^, ^48^, ^49^, ^50^, ^51^, ^52^, ^53^, ^54^, ^55^, ^56^ (Fig. 1) with 12 498 participants were included. The number of participants in individual studies ranged from 35^30^ to 3045.^31^

The distribution of male and female participants in the studies varied, with some studies including only female participants^30^ or only male participants^40^ and others not reporting sex distribution.^38^^,^^50^^,^^52^^,^^53^ The studies matching our inclusion criteria were published between 1982^52^ and 2018,^37^ and assessed postoperative pain after different types of surgical procedures (Table 1). Nine studies included only cognitively intact patients,^6^^,^^32^^,^^35^^,^^38^^,^^47^^,^^49^^,^^51^^,^^54^^,^^55^ whereas two studies included mild cognitively impaired participants.^46^^,^^56^ The remaining 20 studies did not report on cognitive function.^2^^,^^3^^,^^13^^,^^30^^,^^32^, ^33^, ^34^, ^35^, ^36^^,^^39^, ^40^, ^41^, ^42^, ^43^, ^44^, ^45^^,^^48^^,^^50^^,^^52^^,^^53^

**Table 1:** Characteristics of included studies. BNS, box numerical rating scale; CAS, coloured analogue scale; CCPS, colour circle pain scale; ENT, ear, nose and throat; FPS, face pain scale; ICU, intensive care unit; MPQ, McGill pain questionnaire; M-VRS, modified verbal rating scale with 11 description of pain intensity; NR, not reported; NRS, numerical rating scale; OPS, objective pain score; PCA, patient controlled analgesia; PPI, present pain intensity; PROM/s, patient-reported outcome measures; RWS, red wedge scale; sd, standard deviation; THA, total hip arthroplasty; TKA, total knee arthroplasty; VAS-R, visual analogue scale at rest; VAS-M; visual analogue scale at movement; VDS, verbal descriptor scale; VPS, 11-point verbal scale; VRS∗∗, 4-point verbal rating scale; VRS-5, 5-point verbal rating scale; VRS-P; verbal rating scale for pain relief.

First author, year (country)	PROM/s	Study design	Surgical procedure	Outcome(s)	High anchor∗	Main exclusion criteria	Patient characteristics	
							*n* (Female%)	Age (yr) Mean (sd) [range]
Van Dijk, 2015^13^ (The Netherlands)	NRS	Cross-sectional design	Orthopaedic, ENT, gynaecological, cardiothoracic, Others	Ability to detect desire for analgesics	Worst pain imaginable	ICU patients, not proficient in Dutch or English, ambulatory surgery	1084 (48)	53 [18–90]
Banos, 1989^34^ (Spain)	VAS VRS-5	Descriptive correlational design	Abdominal, orthopaedic, gynaecological	Convergent validity	10 Unbearable pain	NR	212 (50)	<30=43 31–50=69 >50=107
Akinpelu, 2002^30^ (Nigeria)	VAS M-VRS BNS	Cross-sectional design	Caesarean section	Convergent validity	Worst pain Worst imaginable Worst pain	Complications, illness unconscious	35 (100)	31 (5)
Briggs, 1999^35^ (UK)	VAS VRS∗∗	Secondary analysis of RCT	Orthopaedic	Convergent validity Feasibility	Number 100 Severe pain at rest and movement	NR	417 (45)	47 (20)∗ 64 (17)
Fadaizadeh, 2009^39^ (Iran)	VAS FPS	Cross-sectional design	General, gynaecological	Convergent validity	10 Agonised	History of substance abuse, unconscious	82 (72) 34 GS 48 GYN	32 (14) GYN 27 (7) GS 38 (18)
Deloach, 1998^38^ (USA)	VAS VPS	Descriptive correlational design	Various type of surgeries	Convergent validity	Worst imaginable Horrible pain	NR	NR	NR
Pesonen, 2008^51^ (Finland)	VAS VRS-5 RWS FPS-7	Descriptive correlational design	Cardiac surgery: elective CABG, valvular repair	Feasibility	Worst possible pain Unbearable pain Worst possible pain Worst possible pain	Dementia, cognitive impairment	160 FPS 80 (36) RWS 80 (44)	73 (5)
Aubrun, 2003^32^ (France)	VAS NRS VRS Behavioural scale	Prospective observational design	Orthopaedic, abdominal, gynaecological, others	Feasibility	Worst imaginable pain Worst imaginable pain Severe NR	NR	600 (47)	51 (17)
Myles, 1999^49^ (Australia)	VAS	Clinical study	General, orthopaedic, ENT, faciomaxillary, cardiothoracic	Interpretability	100 worst pain ever	Severe pain, inability to complete the VAS	52 (40)	42 (15)
Myles, 2005^50^ (Australia)	VAS	Clinical study	General, orthopaedic, ENT, faciomaxillary, cardiothoracic	Interpretability	100 worst pain ever	Postoperative delirium Frailty, visual impairment	22 (NR)	33 (17)
Jensen, 2003^44^ (USA)	VAS VRS-4 VRS-P	Secondary analysis of RCT	Total knee replacement, hysterectomy, laparotomy	Interpretability	Worst pain Severe pain Complete relief	NR	123 (66)	65 (10)
Gerbershagen, 2011^41^ (Germany)	NRS	Comparative study design	Cholecystectomy, thyroidectomy, gastrointestinal, inguinal hernia repair, others	Interpretability	Worst imaginable pain	Repeated surgical, procedures, mechanical ventilation	444 (44)	18–20=38 21–30=75 31–40=88 41–50=96 51–60=87 61–70=49 71–80=2
Cepeda, 2003^36^ (USA)	NRS VRS	Clinical study	Head and neck, thoracic, spinal abdominal, orthopaedic	Interpretability	Worst imaginable Severe pain	NR	700 (62)	50 (15)
Jensen, 2002^45^ (USA)	VAS VRS Pain relief	Secondary analysis of RCT	Total knee replacement, abdominal hysterectomy, laparotomy	Responsiveness	Worst pain Severe pain Complete relief	NR	246 (66)	Knee 65 (10) Laparotomy 41 (7.5)
Jenkinson, 1995^43^ (UK)	VAS CPI McGill	RCT	Orthopaedic	Responsiveness	Severe pain	NR	75 (64)	Male: 41 (13) Female: 43 (12)
Aubrun, 2003^31^ (France)	VAS	Clinical study	Orthopaedic, urological, abdominal gynaecological, vascular, thoracic	Interpretability	100	Minor pain, delirium, dementia, non-French speaking	3045 (54)	50 (18)
Sriwatanakul, 1982^52^ (USA)	VAS	Secondary analysis of RCT	NR	Interpretability	Pain as bad as it could be	NR	NR	NR
Van Giang, 2015^55^ (Vietnam)	FPS NRS	Validation study	Orthopaedic	Concurrent validity Responsiveness	The worst possible pain	Hearing impairment Altered mental status	144 (45)	37 (13)
Van Dijk, 2012^54^ (The Netherlands)	NRS VRS	Cross-sectional design	General, ENT, orthopaedic, neurosurgical, urological, gynaecological, plastic, vascular, cardiothoracic	Interpretability	10 Worst pain imaginable	ICU patients Non-Dutch speaking Cognitive or hearing impairment, inability to use self-report	2674 (51)	73 (6)
Li, 2007^47^ (China)	VAS NRS-11 VDS FPS	Prospective clinical study	NR	Convergent validity Scale reliability Responsiveness Feasibility	10 The most intense imaginable pain 10 The most intense imaginable pain The most intense imaginable pain Worst pain	NR	173 (45)	45.3 (15)
Li, 2009^46^ (China)	FPS NRS IPT	Descriptive correlational design	Gastrointestinal, orthopaedic, abdominal	Convergent validity Scale reliability Responsiveness Feasibility	10 10 The most intense imaginable pain	Did not speak Chinese More than one surgery ASA score of 4 Chronic pain	180 (68)	72 (6)
Zhou, 2011^56^ (China)	VDS NRS FPS CAS	Descriptive comparative design	NR	Criterion validity Convergent validity Test–retest reliability Feasibility	Worst pain	Severe cognitive impairment	200 (46)	56 (16)
Gagliese, 2005^6^ (Canada)	VAS-H VAS-V NRS VDS MPQ	Validation study	NR	Feasibility Convergent validity Criterion validity	10 Worst possible pain 10 Worst pain imaginable Excruciating	On epidural or regional analgesia, ASA score of >3 Chronic pain, cognitive impairment, opioid or substance abuse	504 (58)	53 (15)
Tandon, 2016^53^ (India)	OPS NRS	Descriptive correlational design	Abdominal surgery	Convergent validity	Worst possible pain Inadequate pain relief/pain at rest	Haemodynamic instability Unable to use a PCA pump	93	NR
Aziato, 2015^33^ (Ghana)	NRS FPS CCPS	Two phases: qualitative and psychometric testing	Caesarean section, leg amputation, laminectomy, laparotomy, others	Convergent validity Inter-rater reliability Responsiveness Feasibility	Worst possible pain Hurts worst	NR	150 (77)	<30=44.7 30–39=35 40+=21
Hamzat, 2009^42^ (Ghana)	VAS	Validation study	Various gynaecological procedures	Cross-cultural validity	Worst possible pain	History of psychological or psychiatric disorders	60 (100)	NR
Gagliese, 2003^40^ (Canada)	MPQ PPI VAS-R VAS-M	Descriptive correlation design	Radical prostatectomy	Convergent validity Responsiveness	Worst possible pain 5 Excruciating 10 Worst possible 10 Worst possible pain	Non-English speaker ASA >3 Chronic pain Chronic use of opioids	200	Younger patients: 56 (6) Older patients: 67 (3)
Myles, 2017^48^ (Australia)	VAS	Observational design	General, orthopaedic, gynaecological, urological, major vascular, cardiac faciomaxillary, others	Test–retest reliability Interpretability	Very severe pain	Poor English comprehension Drug or alcohol dependence Psychiatric disorder Uncontrolled pain	219 (68)	53 (17)
Danoff, 2018^37^ (USA)	VAS	Prospective observational design	THA TKA	Measurement error	Worst possible pain	Preoperative pain Catastrophizing Scale score greater than 30 points	304 THA (21) TKA (30)	THA: 60 [20–81] TKA; 63 [46–88]
Sloman, 2006^2^ (Israel)	NRS	One group pretest–post-test design	Abdominal, orthopaedic, others	Interpretability	10 Excruciating	NR	150 (47)	47 [14–89]
Bodian, 2001^3^ (USA)	VAS McGill	Clinical study	Intra-abdominal Surgery	Interpretability Desire for analgesics	Worst pain imaginable	NR	150 (48)	49 [37–61]

∗ The low anchor was “no pain”.

Seven studies were performed in the USA,^3^^,^^36^, ^37^, ^38^^,^^44^^,^^45^^,^^52^ three in China,^46^^,^^47^^,^^56^ three in Australia,^48^, ^49^, ^50^ and two each in the UK,^35^^,^^43^ the Netherlands,^13^^,^^54^ Ghana,^33^^,^^42^ France,^32^ and Canada.^6^^,^^40^ One study each was performed in Finland,^51^ Spain,^34^ Nigeria,^30^ Iran,^39^ India,^53^ Vietnam,^55^ Israel,^2^ and Germany.^41^ Although all the included studies were reported in English, some of the tools were administered in other languages: Chinese,^46^^,^^47^^,^^56^ Twi,^33^^,^^42^ Vietnamese,^55^ Finnish,^51^ and both English and Yoruba.^30^

Using the modified Newcastle–Ottawa Score, the majority of studies looking at feasibility were of medium^2^^,^^30^^,^^32^^,^^33^^,^^37^^,^^39^^,^^49^^,^^54^ or high quality.^3^^,^^6^^,^^13^^,^^35^^,^^36^^,^^41^^,^^46^, ^47^, ^48^^,^^50^^,^^51^ The methodological quality of three secondary analysis studies that looked at VAS interpretability could not be assessed.^44^^,^^45^^,^^52^ The methodological quality for other measurement properties is described under each measurement property section.

The following measurement properties were assessed: measurement error (*n*=1),^37^ cross-cultural validity (*n*=1),^42^ reliability (*n*=5),^33^^,^^46^, ^47^, ^48^^,^^56^ responsiveness (*n*=7),^33^^,^^40^^,^^43^^,^^45^, ^46^, ^47^^,^^55^ and hypothesis testing for construct validity (namely convergent validity; *n*=13)^6^^,^^30^^,^^33^, ^34^, ^35^^,^^38^, ^39^, ^40^^,^^46^^,^^47^^,^^54^, ^55^, ^56^ and criterion validity (*n*=2).^6^^,^^56^ No studies assessed structural validity, internal consistency, or content validity of any pain assessment tool. Interpretability was measured in 11 studies.^2^^,^^3^^,^^31^^,^^36^^,^^41^^,^^44^^,^^48^, ^49^, ^50^^,^^52^^,^^54^ Two studies included the desire for analgesics as an outcome.^3^^,^^13^ The feasibility of pain assessment tools as an outcome measure was examined in eight studies.^6^^,^^32^^,^^33^^,^^35^^,^^46^^,^^47^^,^^51^^,^^56^

### Outcomes for measurement properties

#### Unidimensional pain assessment tools

##### Convergent validity

Eight studies^6^^,^^30^^,^^34^^,^^35^^,^^38^, ^39^, ^40^^,^^47^ reported the convergent validity of the VAS with moderate-to-high correlations between several self-report scales that also measured pain intensity. Similarly, seven studies reported good convergent validity results for VRS,^6^^,^^34^^,^^35^^,^^45^^,^^47^^,^^54^^,^^56^ and six studies each reported good convergent validity results for NRS^6^^,^^33^^,^^46^^,^^47^^,^^54^^,^^56^ and FPS^33^^,^^39^^,^^46^^,^^47^^,^^55^^,^^56^ scores (Table 2). The correlations between scores obtained from several unidimensional tools were moderate to high, ranging from 0.5 to 0.9.

**Table 2:** Summary of methodological quality of studies using COSMIN risk of bias and measurement properties. COSMIN, COnsensus-based Standards for the selection of health Measurement INstruments; FPS, faces pain scale; LoE, Level of evidence using GRADE approach reported as: High, Moderate, Low, or Very low; NRS, numerical rating scale; OBS, objective pain score; VDS, verbal descriptor scale. Ratings for overall quality reported as sufficient (+), insufficient (–), inconsistent (+/–), indeterminate (?). Empty cells indicate no available results for measurement properties.

First author, year	Content validity	Structural validity	Internal consistency	Cross-cultural validity	Reliability	Measurement error	Criterion validity	Construct validity/convergent	Responsiveness
**VAS Methodological quality assessment (COSMIN risk of bias)**									
Banos, 1989^34^								Adequate	
Akinpelu, 2002^30^								Doubtful	
Briggs, 1999^35^								Adequate	
Fadaizadeh, 2009^39^								Adequate	
DeLoach, 1998^38^								Doubtful	
Li, 2007^47^					Inadequate			Adequate	Inadequate
Gagliese, 2005^6^							Inadequate	Inadequate	
Gagliese, 2003^40^								inadequate	Inadequate
Myles, 2017^48^					Inadequate				
Jensen, 2002^45^									Inadequate
Danoff, 2018^37^						Adequate			
Hamzat, 2009^42^				Inadequate					
**Rating** **LoE**				? Very low	+ Low	? Moderate	? Very low	+ High	? Low
**NRS Methodological quality assessment (COSMIN risk of bias)**									
Van Dijk, 2012^54^								Adequate	
Li, 2007^47^					Inadequate			Adequate	Inadequate
Li, 2009^46^					Inadequate			Adequate	Inadequate
Zhou, 2011^56^					Inadequate		Adequate	Adequate	
Gagliese, 2005^6^							Inadequate	Inadequate	
Aziato, 2015^33^					Inadequate			Doubtful	Inadequate
** Rating** **LoE**					+ Low		+/– Low	+ High	? Low
**VDS Methodological quality assessment (COSMIN risk of bias)**									
Banos, 1989^34^								Adequate	
Briggs, 1999^35^								Adequate	
Van Dijk, 2012^54^								Adequate	
Li, 2007^47^					Inadequate			Adequate	
Zhou, 2011^56^					Inadequate		Adequate	Adequate	
Gagliese, 2005^6^							Inadequate	Inadequate	
Jensen, 2002^45^									Inadequate
**Rating** **LoE**					+ Low		+/– Low	+/– High	? Low
**FPS Methodological quality assessment (COSMIN risk of bias)**									
Fadaizadeh, 2009^39^								Adequate	
Van Giang, 2015^55^								Adequate	Doubtful
Li, 2007^47^					Inadequate			Adequate	Inadequate
Li, 2009^46^					Inadequate			Adequate	Inadequate
Zhou, 2011^56^					Inadequate		Adequate	Adequate	
Aziato, 2015^33^					Inadequate			Doubtful	Inadequate
**Rating** **LoE**					+ Low		+ Moderate	+ High	? Low
**OPS Methodological quality assessment (COSMIN risk of bias)**									
Tandon, 2016^53^								Doubtful	
**Rating** **LoE**								+ Very low	

##### Cross-cultural validity

One study^42^ established the validity of a Twi (Ghanaian) version of the VAS. The pain scores reported by patients using the new instrument correlated significantly with those reported by patients using the original (English) version of the VAS, with the highest correlation on the fifth postoperative day. Because of inadequate quality owing to an extremely serious risk of bias and imprecision, very low quality evidence was reported for cross-cultural validity of the VAS.

##### Reliability

The VAS showed high scale,^46^^,^^47^ and test–retest reliability^48^ with an intraclass correlation coefficient of 0.79 (95% confidence interval [CI], 0.49–0.91).^48^ The NRS demonstrated high test–retest,^56^ inter-rater,^44^ and scale reliability.^33^^,^^46^^,^^47^^,^^56^ VDS demonstrated high scale^47^ and test–retest reliability.^56^ Similarly, FPS demonstrated high inter-rater^33^ and test–retest reliability^56^ (Table 3). All four scales showed low-quality evidence because of very serious risk of bias.

**Table 3:** Reliability of unidimensional pain assessment tools in surgical patients. ∗Average interclass correlation coefficient calculated for 7 days. ^†^No separate result for each scale. ^‡^Results categorised in 20–44 yr (*n*=43), 45–59 yr (*n*=39), 60 yr without cognitive impairment (*n*=40), ≤60 yr with mild cognitive impairment (*n*=31). ^¶^95% confidence interval. FPS, faces pain scale; n, number of patients; NRS, numerical rating scale; PROM/s, patient-reported outcome measures; SD, standard deviation; VAS, visual analogue scale; VDS, verbal descriptor scale.

First author, year	PROM/s	Pain construct	Reliability			
			Type	*n*	Time interval	Interclass correlation coefficient
Li, 2007^47^	VAS NRS VDS FPS	Current, worst, least, average pain on 7 postoperative days	Scale reliability	173	Every 24 h	0.66∗ 0.76∗ 0.72∗ 0.72∗
Li, 2009^46^	FPS NRS Iowa Pain Thermometer	Current pain and daily retrospective ratings of worst and least pain	Scale reliability	180	Every 24 h	0.95 to 0.97^†^
Zhou, 2011^56^	VDS NRS FPS Numeric Box-21 Scale Coloured Analogue Scale	Recalled pain and postoperative pain	Test–retest reliability	153	24 h	0.96, 0.88, 0.93, 0.84^‡^ 0.94, 0.90, 0.91, 0.80^‡^ 0.93, 0.91, 0.84, 0.80^‡^ 0.92, 0.91, 0.78, 0.76^‡^ 0.93, 0.90, 0.88, 0.77¶
Aziato, 2015^33^	NRS FPS Colour Circle Pain Scale	No pain – worst possible pain No pain – worst possible pain No pain – unbearable	Inter-rater reliability	150	5–10 min	0.92 0.93 0.93
Myles, 2017^48^	VAS	Pain unchanged or almost the same	Test–retest reliability	22	Not reported	0.79 (0.49–0.91)^¶^

##### Responsiveness

Seven studies^33^^,^^40^^,^^43^^,^^45^, ^46^, ^47^^,^^55^ reported responsiveness results for the four unidimensional pain assessment tools and provided low-quality evidence because of a very serious risk of bias (Table 4). The identified risk of bias was mainly related to the use of inappropriate measures of responsiveness such as effect size and statistical tests used.

**Table 4:** Responsiveness results of unidimensional tools. Empty cells indicate data not available or not assessed. ∗*P*-value is statistically significant at <0.0001. ^†^Knee surgery. ^‡^Laparotomy. ^¶^VAS score. ^§^CPI score. ^||^Time 2 *vs* time 1. ^#^Time 3 *vs* time 1. ^††^Time 4 *vs* time 1. ^‡‡^Time 5 *vs* time. ^¶¶^Results for younger patient split of the sample at the median age of 62 yr. CCPS, colour circle pain scale; CI, confidence interval; CPI, categorical verbal pain rating scale; FPS, face pain scale; G, group; MPQ, McGill pain questionnaire; PPI, present pain intensity; PROM/s, patient-reported outcome measures; SRM, standardized response mean; VAS, visual analogue scale; VAS-R, visual analogue scale at rest; VAS-M, visual analogue scale at movement; VDS, verbal descriptor scale. Effect size, calculated by taking a mean change of variable and dividing it by standard deviation of that variable.

First author, year	PROM/s	Time interval	*n*	Better, same, worse %	Mean difference before and after treatment (95% CI)	Effect size OR SRM (95% CI)	Correlation with changes in other instruments
Jensen, 2002^45^	VAS VDS Relief rating	Baseline then several times	123 125		10.37,^†^ 20.71^‡^ 7.17,^†^ 15.09^‡^ 7.59,^†^ 26,61¶		
Jenkinson, 1995^43^	VAS CPI MPQ	Baseline then 120 min	75	Moderate 2.23,^¶^ 1.83^§^ Good 1.91,^¶^ 3.13^§^ Complete 1.89,^¶^ 5^§^		G1;0.99,^¶^ 1.93^§^ G2;1.23,^¶^ 1.82^§^ G3; 2,^¶^ 3.29^§^ G4;1.48,^¶^ 1.48^§^	CPI 0.67 to VAS
Van Giang, 2015^55^	FPS NRS	Every 30 min for 2 h	144		–1.17^||^ –1.59^#^ –1.66^††^ –1.82^‡‡^	–0.70^||^ –1.05^#^ –1.20^††^ –1.31^‡‡^	0.78
Li, 2007^47^	VAS NRS VDS FPS	NR	28		4.3 [2.4]^††^ 4.2 [2.3]^††^ 4.5 [2.1]^††^ 4.3 [1.9]^††^		
Li, 2009^46^	FPS NRS JPT	NR	180		14.095^|| ††^		
Aziato, 2015^33^	NRS FPS CCPS	NR	150		2.3 (2.1–2.5)^††^ 1.5 (1.4–1.6)^††^ 1.4 (1.3–1.5) ^††^		
Gagliese, 2003^40^	MPQ PPI VAS-R VAS-M	NR	200			0.31,^¶¶^ 0.39 0.25,^¶¶^ 0.26 0.23,^¶¶^ 0.32 Not reported	

##### Measurement error

Only one study assessed measurement error of VAS by determining the minimal detectable change (MDC),^37^ which describes the smallest change outside of inherent measurement error that the VAS can detect. The study showed that the MDC on a 100 mm VAS was 15 mm for total hip arthroplasty and 16 mm for total knee arthroplasty.^37^ We evaluated the evidence regarding VAS measurement error as moderate quality because we could not determine the minimal important change for VAS in acute pain to compare with MDC and the risk of bias.

#### Functional pain assessment tool

Only one study examined the ‘Objective Pain Score’, which assesses the interference of pain with respiratory function.^53^ The study evaluated the correlation between scores obtained from the Objective Pain Score and NRS. Whilst patients rated their pain using a printed NRS, the clinician rated pain using the Objective Pain Score. A linear regression model determined the relationship between NRS and Objective Pain Score, and showed that, for every unit increase in the NRS, the Objective Pain Score decreased by 0.334. The study reported sufficient convergent validity with the NRS, although with low-quality evidence because of risk of bias and imprecision. A summary of findings on all assessed measurement properties is provided (Table 2).

### Other outcomes

#### Interpretability and desire for analgesics

##### Visual analogue scale

Seven studies^31^^,^^37^^,^^44^^,^^48^, ^49^, ^50^^,^^52^ looked at the interpretability of VAS, and one study^3^ included the desire for analgesics as an outcome. Several studies^31^^,^^44^^,^^52^ reported nearly similar cut-off points for VAS, indicating that VAS ratings of 0–5 mm were very likely to be rated as no pain by patients, 6–44 mm were considered mild pain, 45–69 mm were considered moderate pain, and VAS ratings ≥70 mm were suggestive of severe pain.

Two studies^37^^,^^48^ determined the interpretability of VAS by identifying the minimal clinically important difference (MCID) defined as the minimal change in score indicating a meaningful change in pain status.^57^ The use of a combination of distribution- and anchor-based methods resulted in an MCID of 9.9 mm for VAS in assessing several types of surgical procedures.^48^ In contrast, Danoff and colleagues^37^ reported higher MCID values for pain improvement in patients undergoing total hip or knee arthroplasty. Pain was improving clinically when the VAS decreased by 19 and 23 mm, respectively.

Bodian and colleagues^3^ found that the proportion of patients requesting additional analgesia after abdominal surgery increased as VAS increased (4%, 43%, and 80% with VAS scores of 30 mm or less, 31–70 mm, and greater than 70 mm, respectively).

##### Numerical rating scale

Four studies^2^^,^^36^^,^^41^^,^^54^ looked at interpretability of the NRS, and one study included desire for analgesics as an outcome.^13^ Sloman and colleagues^2^ determined the meaning of changes in NRS in relation to perceived pain relief before and after treatment. Patients who rated their pain relief as ‘minimal’ had, on average, a 35% reduction in NRS. NRS was less sensitive to detect changes from ‘moderate’ to ‘much’ as there was a 67% reduction for those who rated their reduction as ‘moderate’, a 70% decrease for those who rated it is as ‘much’, and a 94% reduction for those assessed their pain reduction as ‘complete’.^2^

Inconsistent cut-off points between moderate to severe pain were identified for NRS. For example Gerbershagen and colleagues^41^ determined NRS ≥4 as a cut-point for moderate pain, whereas ‘pain interfering with function’ resulted in a lower cut-off point of NRS ≥3. While using receiver operating characteristic analysis in another study, Van Dijk and colleagues^54^ found that the sensitivity of NRS to differentiate bearable pain (VRS ≤2) from unbearable pain (VRS >2) reached higher values (94%) for high cut-off point of NRS >5 compared with lower cut-off points of 3 and 4 (sensitivity 72% and 83%), respectively.

In another study, Van Dijk and colleagues^13^ showed that 19% of patients with NRS scores ranging from 5 to 10 had no desire for additional opioids; meanwhile, 62% reported that they did not want additional opioids because their pain was tolerable. When patients were asked at which score they would request opioids, both the median and the modal pain scores were an NRS of 8.

#### Feasibility

Eight studies included feasibility of pain assessment tools as an outcome measure.^6^^,^^32^^,^^33^^,^^35^^,^^46^^,^^47^^,^^51^^,^^56^ Error rates were reported as an inability to understand the tool, responses that could not be scored reliably, and lack of responses.^6^^,^^35^^,^^47^^,^^51^ Some studies reported the most preferred scale or the easiest to complete ones.^6^^,^^33^^,^^46^^,^^56^ There was a lack of studies that assessed the time required to complete the tool or time taken to train patients or nurses.

For multiple types of surgical procedures and in different populations, VDS or VRS was more successful when compared with other tools. Using VRS in patients aged ≥75 yr after cardiac surgery showed a higher success rate (81%) compared with VAS (60%) and the FPS (44%). These rates varied significantly on all postoperative days (*P*<0.02).^51^ The reported reasons for the failure rate, which was identified as failure to understand or express level of pain using the assessment tool, were postoperative confusion, delirium, exhaustion, and an inability to differentiate between facial expressions.^51^ In a similar way, VRS was more suited for compliance and ease of use after orthopaedic surgery compared with VAS in which 56% of patients included in the study did not understand how to complete VAS and one-third could not perform the assessment using VAS because of visual or hearing impairment.^35^ Moreover, VAS showed the highest error rate of 12.3% when used in Chinese populations, whereas VRS reported the lowest error rate (0.8%), which was statistically significant (*P*<0.05).^47^ Interestingly, 40% of the patients rated NRS as the easiest, most preferred tool for assessment; in contrast, VAS was reported the least preferred.^6^

From the nurses' perspectives in PACUs, NRS was the most preferred tool in 60% of the included sample.^32^ Even though the VAS was the recommended tool to be used in the institution where the study was conducted, 50% of the nurses preferred to use either NRS or VRS owing to its complexities making it difficult for patients to understand VAS.^32^ Three studies reported FPS as the preferred tool among a Chinese population,^47^ for women,^46^ middle-aged adults, and older patients without and with mild cognitive impairment, followed by VRS and NRS.^56^ Likewise, FPS (55%) was preferred to NRS (33%) among a Ghanaian population.^33^

## Discussion

This systematic review presents a comprehensive examination of the measurement properties of unidimensional and functional assessment tools used for adult postoperative patients. The quality of evidence for the measurement properties and utility of the VAS, VDS, NRS, and FPS was suboptimal. Overall, construct validity (convergent validity) was most commonly assessed across measures. Content validity, internal consistency, and structural validity were not assessed as these measures are not designed for single-item scales. The VAS had the greatest number of studies assessing its measurement properties in the postoperative setting, followed by the NRS. Studies on functional pain assessment tools were scarce. Most of the reviewed studies failed to meet the COSMIN methodological standards required. Good-quality studies were found for interpretability and feasibility as assessed by the Newcastle–Ottawa Scale.^26^

Most of the studies reported sufficient convergent validity of several unidimensional pain assessment tools, indicating that the scales tended to measure score variations in the same direction.^58^ Similar positive findings of good convergent validity results were reported when these tools were used to assess pain associated with rheumatoid arthritis,^59^ osteoarthritis,^60^ and low back pain.^61^ However, the methodology used to measure convergent validity was limited. Because no gold standard tool exists for assessing pain, most studies assessed the correlation of scores obtained from one unidimensional tool with another, measuring only pain intensity. However, when a multidimensional tool such as the McGill Pain Questionnaire was used as a comparator, studies reported lower correlation scores.^6^^,^^40^^,^^62^ This variation may be related to assessor and patient fatigue during the detailed pain assessment.

There was good reliability of pain assessment for all unidimensional tools. However, the quality of evidence was low for all four scales because of serious risk of bias owing to unreported intervals for repeated measures or the use of inappropriate reliability measures by treating ranked NRS, VDS, or FPS scores as a continuous value. Measurement error was only available for VAS; however, the study outcome was indeterminate because we could not determine for VAS in acute pain to compare it with the MDC. When the MDC is smaller than the minimal important change, significant change can be distinguished from measurement error.^63^

Small, albeit statistically significant changes in VAS do not necessarily indicate clinically important changes to guide the interpretation of studies evaluating analgesic therapies.^37^ Therefore, obtaining an accurate MCID is crucial.^64^ Previous studies have shown that the MCID differs by patient population and diagnosis. We identified two studies reporting inconsistent MCID values for the postoperative population.^37^^,^^48^ The MCID tended to be higher in patients who underwent joint arthroplasties than other procedures.^48^ One explanation might be that patients reporting severe, acute pain need a larger reduction in pain to be clinically meaningful.^65^

Measures of responsiveness are an important psychometric property to assess the sensitivity of change in pain over time.^66^ Measures of responsiveness used included effect size, standardized response mean, and scores before and after intervention.^33^^,^^40^^,^^43^^,^^44^^,^^46^^,^^47^^,^^55^ According to COSMIN methodology, effect size and standardised response mean are inappropriate to assess responsiveness because they measure the size of the change scores rather than their validity. Moreover, the *P*-value of statistical tests only measures the statistical significance of the change in scores rather than their validity.^63^

Pain assessment tools help diagnose surgical catastrophes, allow communication between healthcare providers, and are used to assess efficacy of analgesic treatments and allow comparison between therapies. As no agreement exists on how to identify the optimal cut-off point of a unidimensional pain assessment tool, various arbitrarily chosen values are used.^41^ In general, VAS cut-off points of 30, 70, and 100 mm indicate the upper boundaries of mild, moderate, and severe pain, respectively. However, a recent study conducted found a higher cut-off point between mild and moderate pain of around 55 mm on the VAS, which is greater than the values reported by most earlier studies and physicians' consensus.^44^^,^^67^, ^68^, ^69^

NRS cut-off points used by healthcare professionals do not necessarily reflect patients' desire for additional analgesics.^13^ Previous studies have also found that a high proportion of patients with pain scores >4 did not demand analgesics (28% of patients visiting an emergency department^70^ and 42% of children after surgery^71^). Cho and colleagues^62^ showed that postoperative patients requested an analgesic when their pain was VAS ≥5.5, NRS ≥6, FPS-R ≥6, or VRS ≥2 (moderate or severe pain). This might be influenced by a general refusal for analgesic medicines, or fear of side-effects or addiction, especially with opioids.^13^^,^^72^^,^^73^ Cut-off points, although important, are not validated to guide analgesic interventions.

Previously, postoperative pain assessment and management was focused on providing humanitarian pain relief, which constitutes only one objective to tackle a complex experience, and that was achieved by using unidimensional scores. However, healthcare providers should address pain by several approaches to determine if the pain is tolerable, is hindering recovery, or requires intervention.^62^

Efforts have been made to encourage use of multidimensional tools to assess postoperative pain. A recent systematic review indicated that the Brief Pain Inventory and the American Pain Society Pain Outcomes Questionnaire – Revised were the two commonly used and studied multidimensional pain assessment tools for patients after surgery, followed by the McGill Pain Questionnaire. These multidimensional tools showed good ratings for some psychometric properties such as internal consistency. However, this recommendation was based on low- to moderate-quality evidence.^66^ Moreover, these tools involve a detailed assessment that can range from 5 to 30 min,^74^ hindering routine use for frequent assessment in a busy surgical ward.^20^ Alternatively, functional pain assessment has been recommended.^14^^,^^75^

However, as no gold standard objective measures exist for pain-related functional capacity in postoperative patients,^76^ we included objective tools assessing the impact of pain on function. Only one study reported sufficient convergent validity of functional assessment based on pain interference with normal breathing and NRS score.^53^ The low methodological quality of the study limits the generalisability of the result. Other researchers have tried to incorporate a non-formally validated three-level ‘Functional Activity Score’^20^ into clinical practice. One study in a Chinese population combining the Functional Activity Score and dynamic NRS found that this allowed nurses to guide and educate patients to better use patient-controlled analgesia to facilitate functional recovery.^77^ In addition, a pilot study in hospitalised patients validated a four-level scale (no interference, interference with some or most activities, or inability to do any activity).^78^ It established the convergent validity of this tool compared with NRS and VAS in cognitively intact patients. Patients aged ≥40 yr also preferred a functional assessment scale,^78^ possibly because functional assessment considered the impact of pain on activity.

The heterogeneity of study designs, including the assessment scales used, surgical procedures, sample sizes, countries in which the studies were conducted, and the languages used, make determining the most feasible assessment tool difficult. However, the VAS showed the highest error rate and was the least preferred in several studies, whereas the VRS showed the lowest error rate. Difficulties comprehending the VAS and linearly quantifying pain resulted in a higher frequency of incomplete responses, especially for older patients.^12^^,^^13^ Therefore, older adults and children who have less abstract thinking ability might prefer a categorical scale such as the VRS for easier use.^14^ Interestingly, although the FPS is commonly used in paediatric populations, it was also the most preferred tool in the Ghanaian and Chinese adult populations. This might be because of the simplicity of facial expressions, which can quickly reflect pain. Alternatively, cultural aspects may explain why the FPS was preferred.^79^

### Strengths and limitations

The main strength of this review is that it includes the most frequently used unidimensional and functional pain assessment tools. In addition, we put no limits on publication date, enabling us to obtain information on early studies of these tools. To our knowledge, this is the first review to evaluate the validity of these tools, focusing solely on postsurgical populations and applying COSMIN methodology.

Potential limitations include the fact that the search strategy may have excluded grey literature and studies published in languages other than English. However, we tried to limit the effect of language and publication biases by searching the references of included studies. In addition, the clinical diversity and limitations in the methodologies and quality of the included studies, may have reduced the strength of the conclusions.

### Conclusions

This systematic review challenges the validity and reliability of unidimensional tools to quantify pain in adult patients after surgery. Despite their extensive use, no evidence clearly suggests that one tool has superior measurement properties in assessing postoperative pain. Therefore, future studies should be prioritised to assess their validity, reliability, measurement error, and responsiveness using COSMIN methodology. Moreover, adequate quality head-to-head comparison studies are required to assess several unidimensional pain assessment tools alongside other tools covering multiple dimensions of the pain experience. In addition, because promoting function is a crucial perioperative goal, psychometric validation studies of functional pain assessment tools are warranted to identify patients who need additional interventions to promote recovery and improve postoperative pain assessment and management.

## Authors' contributions

Study design: RMB, AI, DNL, RDK, NAL, LST

Literature search: RMB, AI

Data extraction: RMB, AI

Data analysis: RMB, AI

Data interpretation: RMB, AI, DNL, RDK, NAL, LST

Writing of the manuscript: RMB, AI, DNL, RDK, NAL, LST

Critical review: RMB, AI, DNL, RDK, NAL, LST

Approval of submitted manuscript: RMB, AI, DNL, RDK, NAL, LST

Overall supervision: DNL, RDK

## References

[1] Carr D.B., Goudas L.C. (1999). Acute pain. Lancet.

[2] Sloman R., Wruble A.W., Rosen G., Rom M. (2006). Determination of clinically meaningful levels of pain reduction in patients experiencing acute postoperative pain. Pain Manag Nurs.

[3] Bodian C.A., Freedman G., Hossain S., Eisenkraft J.B., Beilin Y. (2001). The visual analog scale for pain. Anesthesiology.

[4] Breivik H., Borchgrevink P.C., Allen S.M. (2008). Assessment of pain. Br J Anaesth.

[5] Ravaud P., Keita H., Porcher R., Durand-Stocco C., Desmonts J., Mantz J. (2004). Randomized clinical trial to assess the effect of an educational programme designed to improve nurses’ assessment and recording of postoperative pain. Br J Surg.

[6] Gagliese L., Weizblit N., Ellis W., Chan V.W.S. (2005). The measurement of postoperative pain: a comparison of intensity scales in younger and older surgical patients. Pain.

[7] Joyce C., Zutshi D., Hrubes V., Mason R. (1975). Comparison of fixed interval and visual analogue scales for rating chronic pain. Eur J Clin Pharmacol.

[8] Jensen M.P., Karoly P., Braver S. (1986). The measurement of clinical pain intensity: a comparison of six methods. Pain.

[9] Ohnhaus E.E., Adler R. (1975). Methodological problems in the measurement of pain: a comparison between the verbal rating scale and the visual analogue scale. Pain.

[10] Le Resche L., Burgess J., Dworkin S. (1988). Reliability of visual analog and verbal descriptor scales for "objective" measurement of temporomandibular disorder pain. J Dent Res.

[11] Wong D.L., Baker C.M. (1988). Pain in children: comparison of assessment scales. Pediatr Nurs.

[12] Coll A.M., Ameen J.R., Mead D. (2004). Postoperative pain assessment tools in day surgery: literature review. J Adv Nurs.

[13] Van Dijk J.F.M., Kappen T.H., Schuurmans M.J., van Wijck A.J.M. (2015). The relation between patients’ NRS pain scores and their desire for additional opioids after surgery. Pain Pract.

[14] Pasero C., Quinlan-Colwell A., Rae D., Broglio K., Drew D. (2016). American Society for Pain Management Nursing position statement: prescribing and administering opioid doses based solely on pain intensity. Pain Manag Nurs.

[15] Chou R., Gordon D.B., de Leon-Casasola O.A. (2016). Management of postoperative pain: a clinical practice guideline from the American pain society, the American Society of Regional Anesthesia and Pain Medicine, and the American Society of Anesthesiologists' committee on regional anesthesia, executive committee, and administrative council. J Pain.

[16] Mularski R.A., White-Chu F., Overbay D., Miller L., Asch S.M., Ganzini L. (2006). Measuring pain as the 5th vital sign does not improve quality of pain management. J Gen Intern Med.

[17] Frasco P.E., Sprung J., Trentman T.L. (2005). The impact of the Joint Commission for Accreditation of Healthcare Organizations pain initiative on perioperative opiate consumption and recovery room length of stay. Anesth Analg.

[18] Vila H., Smith R.A., Augustyniak M.J. (2005). The efficacy and safety of pain management before and after implementation of hospital-wide pain management standards: is patient safety compromised by treatment based solely on numerical pain ratings?. Anesth Analg.

[19] Laycock H.C., Harrop-Griffiths W. (2021). Assessing pain: how and why?. Anaesthesia.

[20] Levy N., Sturgess J., Mills P. (2018). Pain as the fifth vital sign” and dependence on the “numerical pain scale” is being abandoned in the US: why?. Br J Anaesth.

[21] Levy N., Mills P., Rockett M. (2019). Post-surgical pain management: time for a paradigm shift. Br J Anaesth.

[22] Kehlet H. (1994). Postoperative pain relief—what is the issue?. Br J Anaesth.

[23] Van Boekel R.L.M., Vissers K.C.P., van der Sande R., Bronkhorst E., Lerou J.G.C., Steegers M.A.H. (2017). Moving beyond pain scores: multidimensional pain assessment is essential for adequate pain management after surgery. PLoS One.

[24] Liberati A., Altman D.G., Tetzlaff J. (2009). The PRISMA statement for reporting systematic reviews and meta-analyses of studies that evaluate health care interventions: explanation and elaboration. J Clin Epidemiol.

[25] Ouzzani M., Hammady H., Fedorowicz Z., Elmagarmid A. (2016). Rayyan—a web and mobile app for systematic reviews. Syst Rev.

[26] Wells G.A., Shea B., O’Connell D. (2021). http://www.ohri.ca/programs/clinical_epidemiology/oxford.asp.

[27] Prinsen C.A., Mokkink L.B., Bouter L.M. (2018). COSMIN guideline for systematic reviews of patient-reported outcome measures. Qual Life Res.

[28] Terwee C.B., Mokkink L.B., Knol D.L., Ostelo R.W., Bouter L.M., de Vet H.C. (2012). Rating the methodological quality in systematic reviews of studies on measurement properties: a scoring system for the COSMIN checklist. Qual Life Res.

[29] Mokkink L.B., De Vet H.C.W., Prinsen C.A.C. (2018). COSMIN risk of bias checklist for systematic reviews of patient-reported outcome measures. Qual Life Res.

[30] Akinpelu A.O., Olowe O.O. (2002). Correlative study of 3 pain rating scales among obstetric patients. Afr J Med Med Sci.

[31] Aubrun F., Langeron O., Quesnel C., Coriat P., Riou B. (2003). Relationships between measurement of pain using visual analog score and morphine requirements during postoperative intravenous morphine titration. Anesthesiology.

[32] Aubrun F., Paqueron X., Langeron O., Coriat P., Riou B. (2003). What pain scales do nurses use in the postanaesthesia care unit?. Eur J Anaesthesiol.

[33] Aziato L., Dedey F., Marfo K., Avoka Asamani J., Clegg-Lamptey J.N.A. (2015). Validation of three pain scales among adult postoperative patients in Ghana. BMC Nurs.

[34] Banos J.E., Bosch F., Canellas M., Bassols A., Ortega F., Bigorra J. (1989). Acceptability of visual analogue scales in the clinical setting: a comparison with verbal rating scales in postoperative pain. Methods Find Exp Clin Pharmacol.

[35] Briggs M., Closs J.S. (1999). A descriptive study of the use of visual analogue scales and verbal rating scales for the assessment of postoperative pain in orthopedic patients. J Pain Symptom Manage.

[36] Cepeda M.S., Africano J.M., Polo R., Alcala R., Carr D.B. (2003). What decline in pain intensity is meaningful to patients with acute pain?. Pain.

[37] Danoff J.R., Goel R., Sutton R., Maltenfort M.G., Austin M.S. (2018). How much pain is significant? Defining the minimal clinically important difference for the visual analog scale for pain after total joint arthroplasty. J Arthroplasty.

[38] Deloach L.J., Higgins M.S., Caplan A.B., Stiff J.L. (1998). The visual analog scale in the immediate postoperative period. Anesth Analg.

[39] Fadaizadeh L., Emami H., Samii K. (2009). Comparison of visual analogue scale and faces rating scale in measuring acute postoperative pain. Arch Iran Med.

[40] Gagliese L., Katz J. (2003). Age differences in postoperative pain are scale dependent: a comparison of measures of pain intensity and quality in younger and older surgical patients. Pain.

[41] Gerbershagen H.J., Rothaug J., Kalkman C.J., Meissner W. (2011). Determination of moderate-to-severe postoperative pain on the numeric rating scale: a cut-off point analysis applying four different methods. Br J Anaesth.

[42] Hamzat T., Samir M., Peters G. (2009). Development and some psychometric properties of Twi (Ghanaian) version of the visual analogue scale. Afr J Biomed Res.

[43] Jenkinson C., Carroll D., Egerton M. (1995). Comparison of the sensitivity to change of long and short form pain measures. Qual Life Res.

[44] Jensen M.P., Chen C., Brugger A.M. (2003). Interpretation of visual analog scale ratings and change scores: a reanalysis of two clinical trials of postoperative pain. J Pain.

[45] Jensen M.P., Chen C., Brugger A.M. (2002). Postsurgical pain outcome assessment. Pain.

[46] Li L., Herr K., Chen P. (2009). Postoperative pain assessment with three intensity scales in Chinese elders. J Nurs Scholarsh.

[47] Li L., Liu X., Herr K. (2007). Postoperative pain intensity assessment: a comparison of four scales in Chinese adults. Pain Med.

[48] Myles P., Myles D., Galagher W. (2017). Measuring acute postoperative pain using the visual analog scale: the minimal clinically important difference and patient acceptable symptom state. Br J Anaesth.

[49] Myles P.S., Troedel S., Boquest M., Reeves M. (1999). The pain visual analog scale: is it linear or nonlinear?. Anesth Analg.

[50] Myles P.S., Urquhart N. (2005). The linearity of the visual analogue scale in patients with severe acute pain. Anaesth Intensive Care.

[51] Pesonen A., Suojaranta-Ylinen R., Tarkkila P., Rosenberg P.H. (2008). Applicability of tools to assess pain in elderly patients after cardiac surgery. Acta Anaesthesiol Scand.

[52] Sriwatanakul K., Kelvie W., Lasagna L., Calimlim J.F., Weis O.F., Mehta G. (1983). Studies with different types of visual analog scales for measurement of pain. Clin Pharmacol Ther.

[53] Tandon M., Singh A., Saluja V., Dhankhar M., Pandey C.K., Jain P. (2016). Validation of a new “objective pain score” Vs. “numeric rating scale” for the evaluation of acute pain: a comparative study. Anesth Pain Med.

[54] Van Dijk J.F., Kappen T.H., van Wijck A.J., Kalkman C.J., Schuurmans M.J. (2012). The diagnostic value of the numeric pain rating scale in older postoperative patients. J Clin Nurs.

[55] Van Giang N., Chiu H.-Y., Thai D.H., Kuo S.-Y., Tsai P.-S. (2015). Validity, sensitivity, and responsiveness of the 11-face faces pain scale to postoperative pain in adult orthopedic surgery patients. Pain Manag Nurs.

[56] Zhou Y., Petpichetchian W., Kitrungrote L. (2011). Psychometric properties of pain intensity scales comparing among postoperative adult patients, elderly patients without and with mild cognitive impairment in China. Int J Nurs Stud.

[57] Farrar J.T., Young J.P., LaMoreaux L., Werth J.L., Poole R.M. (2001). Clinical importance of changes in chronic pain intensity measured on an 11-point numerical pain rating scale. Pain.

[58] Hjermstad M.J., Fayers P.M., Haugen D.F. (2011). Studies comparing numerical rating scales, verbal rating scales, and visual analogue scales for assessment of pain intensity in adults: a systematic literature review. J Pain Symptom Manage.

[59] Sendlbeck M., Araujo E.G., Schett G., Englbrecht M. (2015). Psychometric properties of three single-item pain scales in patients with rheumatoid arthritis seen during routine clinical care: a comparative perspective on construct validity, reproducibility and internal responsiveness. RMD Open.

[60] Alghadir A.H., Anwer S., Iqbal A., Iqbal Z.A. (2018). Test-retest reliability, validity, and minimum detectable change of visual analog, numerical rating, and verbal rating scales for measurement of osteoarthritic knee pain. J Pain Res.

[61] Chiarotto A., Maxwell L.J., Ostelo R.W., Boers M., Tugwell P., Terwee C.B. (2019). Measurement properties of visual analogue scale, numeric rating scale, and pain severity subscale of the brief pain inventory in patients with low back pain: a systematic review. J Pain.

[62] Cho S., Kim Y.J., Lee M., Woo J.H., Lee H.J. (2021). Cut-off points between pain intensities of the postoperative pain using receiver operating characteristic (ROC) curves. BMC Anesthesiol.

[63] De Vet H.C., Terwee C.B., Mokkink L.B., Knol D.L. (2011).

[64] Wells G., Beaton D., Shea B. (2001). Minimal clinically important differences: review of methods. J Rheumatol.

[65] Tubach F., Ravaud P., Baron G. (2005). Evaluation of clinically relevant states in patient reported outcomes in knee and hip osteoarthritis: the patient acceptable symptom state. Ann Rheum Dis.

[66] Lapkin S., Ellwood L., Diwan A., Fernandez R. (2021). Reliability, validity, and responsiveness of multidimensional pain assessment tools used in postoperative adult patients: a systematic review of measurement properties. JBI Evid Synth.

[67] Boonstra A.M., Preuper H.R.S., Balk G.A., Stewart R.E. (2014). Cut-off points for mild, moderate, and severe pain on the visual analogue scale for pain in patients with chronic musculoskeletal pain. Pain.

[68] Serlin R.C., Mendoza T.R., Nakamura Y., Edwards K.R., Cleeland C.S. (1995). When is cancer pain mild, moderate or severe? Grading pain severity by its interference with function. Pain.

[69] Zelman D.C., Hoffman D.L., Seifeldin R., Dukes E.M. (2003). Development of a metric for a day of manageable pain control: derivation of pain severity cut-points for low back pain and osteoarthritis. Pain.

[70] Blumstein H.A., Moore D. (2003). Visual analog pain scores do not define desire for analgesia in patients with acute pain. Acad Emerg Med.

[71] Voepel-Lewis T., Burke C.N., Jeffreys N., Malviya S., Tait A.R. (2011). Do 0–10 numeric rating scores translate into clinically meaningful pain measures for children?. Anesth Analg.

[72] Gan T., Lubarsky D., Flood E. (2004). Patient preferences for acute pain treatment. Br J Anaesth.

[73] Miaskowski C. (2000). The impact of age on a patient's perception of pain and ways it can be managed. Pain Manag Nurs.

[74] Wilkie D.J., Savedra M.C., Holzemer W.L., Tesler M.D., Paul S.M. (1990). Use of the McGill Pain Questionnaire to measure pain: a meta-analysis. Nurs Res.

[75] Levy N., Quinlan J., El-Boghdadly K. (2021). An international multidisciplinary consensus statement on the prevention of opioid-related harm in adult surgical patients. Anaesthesia.

[76] White P.F., Kehlet H. (2010). Improving postoperative pain management: what are the unresolved issues?. Anesthesiology.

[77] Tong Y.G., Konstantatos A.H., Yan C., Ling C. (2018). Improving pain management through addition of the functional activity score. Aust J Adv Nurs.

[78] Halm M., Bailey C., St Pierre J. (2019). Pilot evaluation of a functional pain assessment scale. Clin Nurse Spec.

[79] Pasero C., McCaffery M. (2010).

