# Supplementary material for: Utility of unidimensional and functional pain assessment tools in adult postoperative patients: a systematic review
Source: Br J Anaesth. 2022 Jan 5;128(5):874–88. doi: 10.1016/j.bja.2021.11.032 (PMC9074792; doi:10.1016/j.bja.2021.11.032)
Supplement: Multimedia component 1 [file mmc1.docx]

SUPPLEMENTARY DATA

APPENDIX S1: Search strategy

**Search strategy for Ovid Medline** Version 15/08/20

**PICO**

**Population**

Postoperative patients aged 18 years and over from all surgical disciplines.

**Intervention**

Unidimensional pain assessment tools including

- Verbal or printed numerical pain rating scale.
- Printed or verbal descriptor scale.
- Visual analogue scale.
- Faces scales: Wong-baker FACES, Faces Pain Scale – Revised.

Functional pain assessment tools

**Comparison**: -------

**Outcomes**: psychometric properties including validity and reliability

**Additional outcomes**

Instrument feasibility, interpretability, and ability to detect desire of analgesia.

Search concepts to be combined for Boolean AND, and used for unidimensional pain assessment tool and then repeated for functional pain assessment tools

1. Outcome terms
2. Pain assessment tool terms
3. Construct: acute postoperative pain
4. 1 AND 2 AND 3
5. 4 + Limits ( english , humans, adults > 18 years)

Did not apply limits full text, abstracts this might include bias in the results

Ovid MEDLINE(R) ALL < 1946 to August 15, 2022>

1 exp PSYCHOMETRICS/ or psychometr*.mp. or measurement propert*.mp. or Validity.mp. or valid*.mp. or exp Validation Study/ or convergent validity.mp. or construct validity.mp. or content validity.mp. or criterion validity.mp. or reliab*.mp. or unreliab*.mp. or Comparative Study.mp. or Feasibility.mp. or Generalizability.mp. or generalisa*.mp. or interpretab*.mp. or Sensitiv*.mp. or Responsive*.mp. or 'Measurement Accuracy'.mp. or 'ease of use'.mp. or Analgesi* response.mp. or 'desire of analgesi*'.mp. or 'Request of analgesic*'.mp. or 'hypotheses testing'.mp. or 'measurement error*'.mp. or Internal consistency.mp. or Data accuracy.mp. or 'standard error of measurement'.mp. [mp=title, abstract, original title, name of substance word, subject heading word, floating sub-heading word, keyword heading word, organism supplementary concept word, protocol supplementary concept word, rare disease supplementary concept word, unique identifier, synonyms] 4890505

2 (pain scale* or pain rating scale* or (pain assessment and (instrument* or tool*)) or pain intensity scale* or pain measurement instrument* or Pain score* or pain intensity assessment).mp. or exp Pain Measurement/ [mp=title, abstract, original title, name of substance word, subject heading word, floating sub-heading word, keyword heading word, organism supplementary concept word, protocol supplementary concept word, rare disease supplementary concept word, unique identifier, synonyms] 113996

3 Visual Analog Scale.mp. or exp Visual analog? Pain scale/ or (visual analog? and (scale or score)).mp. or vas.mp. [mp=title, abstract, original title, name of substance word, subject heading word, floating sub-heading word, keyword heading word, organism supplementary concept word, protocol supplementary concept word, rare disease supplementary concept word, unique identifier, synonyms] 146135

4 ((numeric* and rating and (scale or score)) or numeric scale or nrs or nprs).mp. or exp numerical pain rating scale/ [mp=title, abstract, original title, name of substance word, subject heading word, floating sub-heading word, keyword heading word, organism supplementary concept word, protocol supplementary concept word, rare disease supplementary concept word, unique identifier, synonyms] 26611

5 exp verbal descriptor scale/ or Vds.mp. or exp verbal rating scale*/ [mp=title, abstract, original title, name of substance word, subject heading word, floating sub-heading word, keyword heading word, organism supplementary concept word, protocol supplementary concept word, rare disease supplementary concept word, unique identifier, synonyms] 1128

6 exp face* pain scale*/ or exp wong baker Face*/ or wong baker face*.mp. or exp faces pain scale revised/ or faces pain scale revised.mp. [mp=title, abstract, original title, name of substance word, subject heading word, floating sub-heading word, keyword heading word, organism supplementary concept word, protocol supplementary concept word, rare disease supplementary concept word, unique identifier, synonyms] 594

7 (pain activity assessment or functional pain assessment scale or functional activity score*or functional pain activity scale* or functional assessment tool or objective pain score* or movement evoked pain assessment or assessment of pain at movement or objective pain assessment or clinically aligned pain assessment tool).mp. [mp=title, abstract, original title, name of substance word, subject heading word, floating sub-heading word, keyword heading word, organism supplementary concept word, protocol supplementary concept word, rare disease supplementary concept word, unique identifier, synonyms] 252

8 exp Pain, Postoperative/ or exp acute pain/ or post surgical pain.mp. or surgical pain.mp. or pain post procedure.mp. [mp=title, abstract, original title, name of substance word, subject heading word, floating sub-heading word, keyword heading word, organism supplementary concept word, protocol supplementary concept word, rare disease supplementary concept word, unique identifier, synonyms] 46322

9 1 and 3 and 8 5987

10 1 and 4 and 8 556

11 1 and 5 and 8 6

12 1 and 6 and 8 56

13 1 and 7 and 8 32

14 limit 9 to (Elanguage and humans and "all adult (19 plus years)") 4358

15 limit 10 to (English language and humans and "all adult (19 plus years)") 537

16 limit 11 to (English language and humans and "all adult (19 plus years)") 6

17 limit 12 to (English language and humans and "all adult (19 plus years)") 12

18 limit 13 to (English language and humans and "all adult (19 plus years)") 2

Search strategy for other databases can be provided on demand from the corresponding author

APPENDIX S2: Measurment properties included in the main domians of the COSMIN taxonomy

| **Domain** | **Psychometric property** | **Definition** |
| --- | --- | --- |
| Reliability | Internal consistency  Reliability  Measurement error | The extent that the measurement is free from measurement error such that scores for patients who have not changed are the same under repeated measurements  The extent that items are inter-related  The proportion of the total variance in the measurements that is due to ‘true’ differences between patients (as opposed to error)  Error in a participant’s score that is not attributed to the construct being measured |
| Validity | Content validity  Face validity  Construct validity  Structural validity  Hypothesis testing  Cross-cultural validity  Criterion validity | The extent that an assessment measures what it aims to measure  The extent that an assessment’s content reflects the construct being measured  The extent that an assessment looks like it reflects the construct being measured  The extent that an assessment’s scores are consistent with hypotheses based on the assumption that the tool measures what it purports to measure  The extent that an assessment’s scores reflect the dimensionality of the construct being measured  Construct validity for the items of an assessment  The extent that items on a translated or culturally modified assessment reflect the original items  The extent that an assessment’s scores represent the ‘gold standard’ |
| Responsiveness |  | An assessment and/or it’s items’ ability to detect change over time in the construct being measured |
| Interpretability* |  | The extent that clinical or everyday understanding can be applied to an assessment’s scores |
| Feasibility* |  | How easily a pain measure can be scored and interpreted |

COSMIN, COnsensus-based Standards for the selection of health Measurement INstruments. Adopted from Mokkink LB, et al.^1^ *Interpretability and *feasibility are not considered measurement properties, but important characteristics of a measurement instrument.

APPENDIX S3: Studies ineligible following full-text review

Full paper examined: 38/ Exclusion after complete paper screening 19 papers.

**Excluded papers:**

1. Arnstein P, Gentile D, Wilson M. validating the functional pain scale for hospitalized adults. *Pain Manag Nurs*. 2019; **20:** 418-24.

**Explanation:** Paper validating functional scale for hospitalized chronic pain patient but did not report separte result for surgical patients.

**Reason for exclusion:** No separate results for postoperative pain assessment.

1. Barber MD, Janz N, Kenton K, et al. Validation of the surgical pain scales in women undergoing pelvic reconstructive surgery. *Female Pelvic Med Reconstr Surg*. 2012; **18:** 198-204.

**Explanation:** Surgical pain scale looked at long term functional outcome following surgery.

**Reason for exclusion:** Patients not assessed as inpatients/irrelevant outcome.

1. McCarthy Jr M, Chang CH, Pickard AS, et al. Visual analog scales for assessing surgical pain. *Jl Amn Coll Surg*. 2005; **201:** 245-52.

**Reason for exclusion:** Patients not assessed as inpatients or irrelevant outcome.

1. Blumstein HA, Moore D. Visual analog pain scores do not define desire for analgesia in patients with acute pain. *Acad Emerg Med.* 2003; **10:** 211-4.

**Explanation**: VAS to detect desire of analgesia in acute emergency pain.

**Reason for exclusion**: Not surgical population.

1. Chiu LYL, Sun T, Ree R, et al. The evaluation of smartphone versions of the visual analogue scale and numeric rating scale as postoperative pain assessment tools: a prospective randomized trial. *Can J Anesth*. 2019; **66:** 706-15.

**Reason for exclusion:** Comparison between NRS smart version with paper version.

1. Neudecker J, Raue W, Schwenk W. High correlation but inadequate point-to-point agreement, between conventional mechanical and electronical visual analogue scale for assessment of acute postoperative pain after general surgery. *Acute Pain*. 2006; **8:** 175-80.

**Reason for exclusion:** Comparison between electronic and mechanical VAS.

1. Erden S, Karadag M, Guler Demir S, et al. Cross-cultural adaptation, validity, and reliability of the Turkish version of revised American Pain Society patient outcome questionnaire for surgical patients. *Agri*. 2018; **30:** 39-50.

**Reason for exclusion**: Multidimensional tool (Revised American Pain Society Patient Outcome Questionnaire).

1. Keawnantawat P, Thanasilp S, Preechawong S. Translation and validation of the Thai version of a modified brief pain inventory: a concise instrument for pain assessment in postoperative cardiac surgery. *Pain Pract*. 2017; **17:** 763-73.

**Reason for exclusion**: Multidimensional tool (modified brief pain inventory).

1. Mendoza TR, Chen C, Brugger A, et al. The utility and validity of the modified Brief Pain Inventory in a multiple-dose postoperative analgesic trial. *Clin J Pain*. 2004; **20:** 357-62.

**Reason for exclusion:** Multidimensional tool (Brief Pain Inventory).

1. Mwachiro M, Mwachiro E, Wachu M, et al. assessing post-operative pain with self-reports via the Jerrycan Pain Scale in Rural Kenya. *World J Surg*. 2020; **44:** 3636-42.

**Reason for exclusion**: Applicability of irrelevant tool (Jerrycan Pain Scale).

1. Jain R, Grewal A. A randomized comparative study assessing efficacy of pain versus comfort scores. *Saudi J Anaesth*. 2017; **11**: 396-401.

**Reason for exclusion**: Retracted paper.

1. Liu WH, Aitkenhead AR. Comparison of contemporaneous and retrospective assessment of postoperative pain using the visual analogue scale. *Br J Anaesth*. 1991; **67**: 768-71.

**Reason for exclusion**: Irrelevant outcome.

1. Salo D, Eget D, Lavery RF, Garner L, Bernstein S, on K. Can patients accurately read a visual analog pain scale? *Am J Emerg Med*. 2003; **21**: 515-9.

**Reason of exclusion**: Not surgical population.

1. Sills ES, Genton MG, Walsh APH, Wehbe SA. Who's asking? Patients may under-report postoperative pain scores to nurses (or over-report to surgeons) following surgery of the female reproductive tract. *Arch Gynecol Obstet*. 2009; **279**: 771-4.

**Explanation:** Looked at how patient communicate pain between nurse and physician.

**Reason for exclusion**: Irrelevant outcome.

1. Rothaug J, Weiss T, Meissner W. How simple can it get? Measuring pain with NRS items or binary items. *Clin J Pain*. 2013; **29**: 224-32.

**Explanation**: They used different answer format for (binary yes/no answers vs. NRS) in a subset of patients using Quality Improvement in Postoperative Pain Management (QUIPS).

**Reason for exclusion**: Multidimensional tool (QUIPS).

1. Zalon ML. Comparison of pain measures in surgical patients. *J Nurs Meas*. 1999; **7:** 135-52.

**Explanation**: This study aimed to establish the validity of brief pain inventory short form.

**Reason for exclusion**: Validation of multidimensional scale.

1. Halm M, Bailey C, St Pierre J, et al. Pilot evaluation of a functional pain assessment scale. *Clin Nurse Spec*. 2019; **33:** 12-21.

**Explanation**: Sample from medical/surgical, critical care, and rehabilitation units experiencing acute or chronic pain.

**Reason for exclusion**: No separate results for acute postoperative pain.

1. Martin WJJM, Ashton-James CE, Skorpil NE, et al. What constitutes a clinically important pain reduction in patients after third molar surgery? *Pain Res Manag*. 2013; **18:** 319-22.

**Reason for exclusion**: Dental surgery, not hospitalized patients.

1. Rago R, Forfori F, Materazzi G, et al. Evaluation of a preoperative pain score in response to pressure as a marker of postoperative pain and drugs consumption in surgical thyroidectomy. *Clin J Pain*. 2012; **28:** 382-6.

**Reason for exclusion:** Sensitivity of preoperative vas scores after tourniquet pressure inflation.

APPENDIX S4: Newcastle-Ottawa Quality Assessment Scale

**(adapted for cross sectional studies)**

This scale has been adapted from the Newcastle-Ottawa Quality Assessment Scale for cohort studies to perform a quality assessment of cross-sectional studies for the systematic review.

**Selection:** (Maximum 4 stars)

**1) Representativeness of the sample:**

a) Truly representative of the average in the target population. * (all subjects or random sampling)

b) Somewhat representative of the average in the target population. * (non-random sampling)

c) Selected group of users.

d) No description of the sampling strategy.

**2) Sample size:**

a) Justified and satisfactory. (by reporting appropriate sample size calculation) *

b) Not justified.

**3) Non-respondents: (adopted to details about patient refused assessment and reasons are described)**

a) Comparability between assessed and non-assessed is established *

b) The response rate is unsatisfactory, or the comparability between respondents and non-respondents is unsatisfactory. removed

c) No description of the number and reason for refusing assessment.

**4) Ascertainment of the assessment (risk factor):**

a) Validated measurement tool. **

b) Non-validated measurement tool, but the tool is available or described. *

c) No description of the measurement tool.

**Comparability:** (Maximum 2 stars)

1) The subjects in different outcome groups are comparable, based on the study design or analysis. Confounding factors are controlled.

a) The study controls for the most important factor (select one). *

b) The study control for any additional factor. *

**Outcome:** (Maximum 3 stars)

**1) Assessment of the outcome:**

a) Independent blind assessment. **

b) Record linkage. **

c) Self report. *

d) No description.

**2) Statistical test:**

a) The statistical test used to analyse the data is clearly described and appropriate, and the measurement of the association is presented, including confidence intervals and the probability level (p value). *

b) The statistical test is not appropriate, not described or incomplete.

APPENDIX S5: Updated criteria for Good Measurement Properties

| Measurement property | Rating | Criteria |
| --- | --- | --- |
| Reliability | +  ?  - | ICC or weighted Kappa ≥ 0.70  ICC or weighted Kappa not reported  ICC or weighted Kappa < 0.70 |
| Measurement error | +  ?  - | Smallest detectable change (SDC) or limits of agreement (LoA) < minimal important change (MIC)  MIC not defined  SDC or LoA > MIC |
| Hypotheses testing for construct validity | +  ?  - | The result is in accordance with the hypothesis  No hypothesis defined (by the review team)  The result is not in accordance with the hypothesis |
| Cross-cultural validity/ measurement invariance | +  ?  - | No important differences found between group factors (such as age, gender, language) in multiple group factor analysis OR no important DIF for group factors (McFadden’s R < 0.02)  No multiple group factor analysis OR DIF analysis performed  Important differences between group factors OR DIF was found |
| Criterion validity | +  ?  - | Correlation with gold standard ≥ 0.70 OR AUC ≥ 0.70  Not all information for ‘+’ reported  Correlation with gold standard < 0.70 OR AUC < 0.70 |
| Responsiveness | +  ?  - | The result is in accordance with the hypothesis OR AUC ≥ 0.70  No hypothesis defined (by the review team)  The result is not in accordance with the hypothesis OR AUC < 0.70 |

Adapted from Prinsen CA, et al.^2^ then modified by removing structural validity and internal consistency item.

APPENDIX S6: Modified GRADE approach for grading the quality of evidence

| Quality of evidence | Lower if |
| --- | --- |
| High  Moderate  Low  Very low | Risk of bias  −1 Serious  −2 Very serious  −3 Extremely serious |
|  | Inconsistency  −1 Serious  −2 Very serious |
|  | Imprecision  −1 total n = 50–100  −2 total n < 50 |
|  | Indirectness  −1 Serious  −2 Very serious |

The starting point is the assumption that the evidence is of high quality. The quality of evidence is subsequently downgraded with one or two levels for each factor (i.e., risk of bias, inconsistency, imprecision, indirectness) to moderate, low, or very low when there is risk of bias (low study quality), (unexplained) inconsistency in results, or indirect results.^3^ Information on how to downgrade is described in detail in the COSMIN user manual.^1^ n = sample size.

Appendix S7. Definition of quality levels

| Quality Level | Definition |
| --- | --- |
| High | We are very confident that the true measurement property lies close to that of the estimate of the measurement property |
| Moderate | We are moderately confident in the measurement property estimate: the true measurement property is likely to be close to the estimate of the measurement property, but there is a possibility that it is substantially different |
| Low | Our confidence in the measurement property estimate is limited: the true measurement property may be substantially different from the estimate of the measurement property |
| Very low | We have very little confidence in the measurement property estimate: the true measurement property is likely to be substantially different from the estimate of the measurement property |

These definitions were adapted from the GRADE approach.^4^ Information on how to downgrade is described in detail in the COSMIN user manual.^1^

**REFERENCES**

1. Mokkink LB, Prinsen C, Patrick DL, et al. COSMIN methodology for systematic reviews of patient-reported outcome measures (PROMs). *User manual* 2018. https://cosmin.nl/wp-content/uploads/COSMIN-syst-review-for-PROMs-manual_version-1_feb-2018.pdf

2. Prinsen CA, Mokkink LB, Bouter LM, et al. COSMIN guideline for systematic reviews of patient-reported outcome measures. *Qual Life Res*. 2018; **27**: 1147-57.

3. Guyatt G, Oxman AD, Akl EA, et al. GRADE guidelines: 1. Introduction—GRADE evidence profiles and summary of findings tables. *J Clin Epidemiol*. 2011; **64**: 383-94.

4. Schünemann H, Brozek J, Guyatt G, Oxman A, editors. GRADE handbook for grading quality of evidence and strength of recommendations. Updated October 2013. https://training.cochrane.org/resource/grade-handbook
